# Development of a nomogram for predicting extramural venous invasion in colon cancer using spectral CT multiparameter imaging

**DOI:** 10.1186/s40644-026-01098-6

**Published:** 2026-07-31

**Authors:** Wen Zhao, Zhaowu Shen, Gangjing Ge, Xiaoying Liu, Chao Peng, Fang Rui, Cong Huang, Haiyan Yang, Rui Zhang, Jie Jiang

**Affiliations:** 1 Department of Medical Imaging, The First Affiliated Hospital of Kunming Medical University, Kunming, Yunnan Province 650032 China; 2 Department of Radiotherapy, The First Affiliated Hospital of Kunming Medical University, Kunming, Yunnan Province 650032 China; 3 Department of Radiology, Joint Logistics Support Force of PLA, No. 926 Hospital, No. 147 Jianmin Rd, Kaiyuan, Yunnan Province 661699 China; 4 Department of Radiology, The People’s Hospital of Chuxiong Yi Autonomous Prefecture, Chuxiong, Yunnan Province 675099 China

**Keywords:** Colon cancer, Computed tomography, Spectral CT, Extramural venous invasion

## Abstract

**Purpose:**

To develop a predictive model based on spectral CT-derived parameters for preoperative assessment of extramural venous invasion (EMVI) in patients with colon cancer.

**Methods:**

This retrospective study included 235 patients with pathologically confirmed colon cancer from two medical centers between September 2022 and July 2024 (192 from center 1 and 43 from center 2). Virtual monoenergetic images (VMIs) ranging from 40 to 120 keV (20 keV increments) were evaluated to identify the optimal energy level based on tumor CT value, signal-to-noise ratio (SNR), and contrast-to-noise ratio (CNR). On the optimal VMI, spectral CT quantitative parameters were measured in the arterial, venous, and delay phases, and dynamic multiphase parameters were calculated. Patients from center 1 were randomly divided into a training cohort and an internal testing cohort at a 7:3 ratio. The nomogram model was developed using the training cohort and subsequently evaluated in the internal testing cohort and an independent external validation cohort from center 2.

**Results:**

(1) The 60 keV VMI demonstrated superior image quality, showing significantly higher tumor CT value, SNR, and CNR compared with other monoenergetic images (all *P* < 0.05). (2) Compared with EMVI negative patients, positive patients exhibited significantly higher values of ΔHU40 _(delay−arterial)_, ΔHU60 _(delay−arterial)_, ΔIC _(delay−arterial)_, delay enhancement fraction (DEF), and ΔZeff _(venous−arterial)_ (all *P* < 0.05). Multivariable analysis further identified these five parameters as independent predictors of EMVI. (3) A nomogram was constructed based on these parameters and demonstrated AUCs of 0.93, 0.91, and 0.84 in the training, testing, and external validation cohorts, respectively.

**Conclusions:**

The 60 keV VMI provides improved lesion delineation in colon cancer. A nomogram incorporating dynamic multiphase spectral CT parameters enables accurate preoperative prediction of EMVI and may support individualized clinical decision-making.

**Supplementary Information:**

The online version contains supplementary material available at https://doi.org/10.1186/s40644-026-01098-6.

## Introduction

Colorectal cancer remains one of the most prevalent and lethal malignancies in the adult population. In 2024, approximately 152,810 new cases of colorectal cancer were diagnosed in the United States, with colon cancer accounting for nearly two-thirds of cases [1, 2]. Local recurrence and distant metastasis are the principal causes of mortality in patients with colon cancer. Notably, postoperative local recurrence occurs in up to 30% of patients and is strongly associated with tumor growth patterns and surgical strategy [3]. Extramural venous invasion (EMVI) is defined as tumor extension beyond the muscularis propria into extramural veins and represents a key pathological marker of aggressive tumor behavior in colorectal cancer. It has been consistently associated with advanced local invasion, lymph node metastasis, distant dissemination, and postoperative recurrence [4, 5]. A study has shown that patients with EMVI positive colorectal cancer exhibit significantly lower 5-year overall survival than those with EMVI negative disease [6]. Accurate preoperative detection of EMVI is therefore crucial for risk stratification, individualized therapeutic planning, and prognostic assessment.

EMVI is primarily diagnosed based on postoperative histopathology, which restricts the opportunity for making individualized treatment strategies before surgery. In recent years, accumulating evidence has suggested that preoperative imaging assessment may contribute to improved prognostic stratification in patients with colon and rectal cancer [5, 7]. Although high-resolution MRI is the reference standard for local staging and EMVI assessment in rectal cancer [8], its application in colon cancer is limited by anatomical variability and motion artifacts related to bowel peristalsis. According to the National Comprehensive Cancer Network (NCCN) guidelines, enhanced CT is the preferred imaging modality for preoperative staging and EMVI evaluation in colon cancer [9]. Conventional enhanced CT has limited diagnostic performance for EMVI, with a reported sensitivity of approximately 49% and specificity of 77%, leading to a considerable number of EMVI positive cases being overlooked before surgery [10]. These limitations are primarily caused by peritumoral inflammation, desmoplastic reaction, and nearby structural interference, along with the absence of quantitative evaluation standards [11].

Spectral CT can generate multiple quantitative parameters in a single scan, including virtual monoenergetic images (VMIs), iodine concentration (IC), effective atomic number (Zeff), and electron density (ED). These parameters provide a noninvasive and quantitative reflection of tumor microvascular perfusion, stromal composition, and microenvironmental heterogeneity [12]. Previous studies have demonstrated significant associations between spectral CT parameters and tumor differentiation as well as vascular invasion status in colorectal cancer, suggesting a potential biological relationship between quantitative spectral features and aggressive pathological phenotypes [13]. However, few studies have specifically focused on colon cancer. Spectral CT parameters have been shown to facilitate the preoperative evaluation of histological subtype and cellular proliferation in gastric cancer [14], further suggesting their potential sensitivity to tumor angiogenesis and tissue composition differences and providing a theoretical basis for investigating EMVI status. In addition, VMIs enhances lesion conspicuity by improving image contrast and reducing noise, enabling clearer delineation of tumor margins and more accurate reflection of intratumoral heterogeneity [15].

Therefore, this study aimed to address two objectives: (1) to determine the optimal VMI energy level for tumor delineation and assessment of tumor heterogeneity; (2) to evaluate spectral CT-derived parameters as noninvasive imaging biomarkers for preoperative prediction of EMVI in colon cancer, with the goal of providing a quantitative tool to support individualized treatment planning.

## Materials and methods

This retrospective study was approved by the Institutional Review Board of the First Affiliated Hospital of Kunming Medical University (IRB No. 2022-L-112) and conducted in accordance with the Declaration of Helsinki. The requirement for written informed consent was waived.

### Study participants

**Fig. 1:**
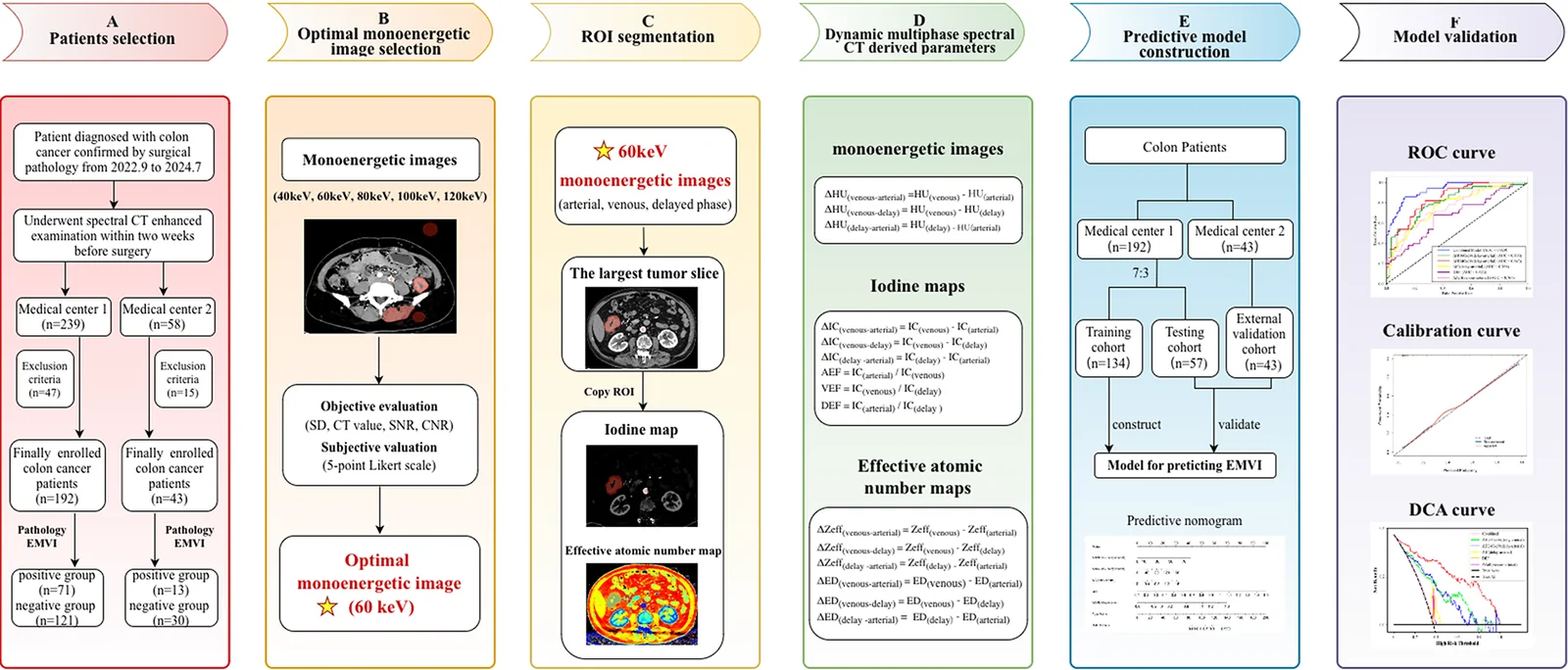
Flowchart of patient enrollment and the study process

Patients with histopathologically confirmed colon cancer who underwent preoperative spectral CT between September 2022 and July 2024 were retrospectively identified from two medical centers: the First Affiliated Hospital of Kunming Medical University (Center 1) and the People’s Hospital of Chuxiong Yi Autonomous Prefecture (Center 2). The inclusion criteria were as follows: (1) pathologically confirmed as colon cancer; (2) pathology report clearly states the presence or absence of extramural venous invasion; (3) underwent spectral CT enhanced examination within two weeks before surgery. The exclusion criteria were as follows: (1) mucinous adenocarcinoma of the colon; (2) presence of synchronous malignancies in other organs; (3) history of prior malignancy; (4) prior gastrointestinal surgery for any reason; (5) CT images with artifacts that interfered with diagnostic interpretation. All eligible patients who met the inclusion and exclusion criteria during the study period were consecutively enrolled from the participating centers. Finally, 192 patients (male: *n* = 107; age: 29–90 years) were enrolled from Center 1, and 43 patients (male: *n* = 22; age: 27–83 years) were enrolled from Center 2. The flowchart of patient enrollment is shown in Fig. 1A.

### Spectral CT examinations and post-processing

Spectral CT (Philips IQon Spectra, Philips Healthcare, Netherlands) was performed for both unenhanced and contrast-enhanced scans. The scanning range extended from the diaphragm to the level of the bilateral ischial tuberosities. The scanning parameters were as follows: tube voltage, 120 kV; tube current, automatically adjusted with exposure control technology; pitch, 1.016; collimator width, 64 × 0.625 mm; rotation time, 0.5 s/rotation; scan slice thickness, 5 mm; reconstruction layer thickness, 1 mm. For the contrast-enhanced scan, a dual-barrel high-pressure injector was used to administer a nonionic contrast agent, iohexol (Bayer HealthCare, 350 mg/mL), via the antecubital vein at a dose of 1.2 mL/kg and a flow rate of 3.0–3.5 mL/s. Contrast enhancement was performed using smart tracking threshold technology, with the trigger region set at the abdominal aorta and a threshold of 140 HU. The arterial phase was scanned at 9 s, the venous phase at 30 s, and the delay phase at 90 s. Images were post-processed using IntelliSpace Portal software (version 10.1, Philips Healthcare) to generate VMIs (40–120 keV, in 20–keV increments), iodine maps, effective atomic number images, and electron density images.

### Image quality in different monoenergetic CT images

The quality assessment process in different VMI CT images is shown in Fig. 1B. Each CT image was measured three times, and the average CT values and standard deviations (SD) were used for analysis. Regions of interest (ROIs) were delineated on the largest slice of the colon tumor on VMIs at the arterial, venous, and delay phases. The ROIs included the largest tumor slice (manually delineated along the tumor boundary), paravertebral back muscles (contoured along the muscle boundary), the abdominal aorta or iliac artery (1 cm²), subcutaneous back fat (12 cm²), and anterior air (20 cm²), as shown in Supplementary Figure 1. Signal-to-noise ratio (SNR) and contrast-to-noise ratio (CNR) were calculated: SNR _tumor_ = CT _tumor_ / SD _tumor;_ CNR _tumor_ = (CT _tumor −_ CT _muscle_) / SD _fat_. Subjective image quality scores were independently evaluated by two experienced abdominal radiologists with 12 and 9 years of experience, respectively, in a double-blind manner. The CT window width and level were set at 400 HU and 40 HU, respectively, to optimize visualization of the lesion and adjacent small vessels. Image noise and overall image quality were assessed using a 5-point Likert scale, with the following criteria: 5 = excellent, 4 = good, 3 = moderate, 2 = fair, and 1 = poor. A score of 3 or higher was considered adequate for clinical diagnostic.

### Spectral CT-derived parameters and dynamic multiphase parameters

**Fig. 2:**
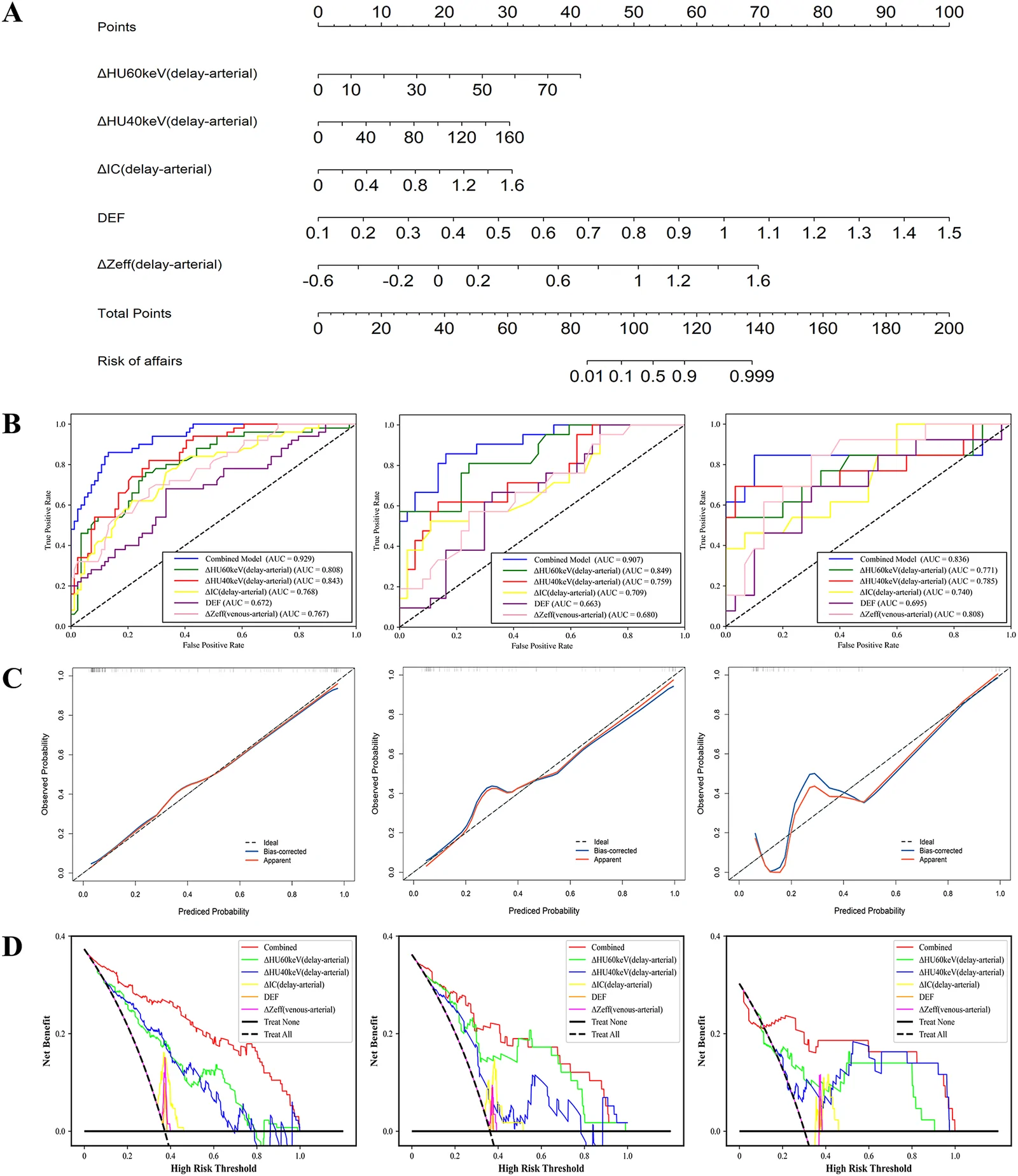
Nomogram for predicting EMVI in colon cancer patients. The nomogram (**A**) was developed with ΔHU60 _(delay−arterial)_, ΔHU40 _(delay−arterial)_, ΔIC _(delay−arterial)_, DEF, and ΔZeff _(venous−arterial)_. (**B**) ROC curve of the nomogram in the training, testing and external validation cohorts respectively. (**C**) Calibration curves of the nomogram for predicting EMVI in the training, testing, and external validation cohorts respectively. (**D**) Decision curve analysis (DCA) of the nomogram for predicting EMVI in the training, testing, and external validation cohorts

Measurements were performed on spectral CT-derived images by two radiologists with expertise in abdominal imaging (12 and 9 years of experience, respectively). The VMI with the optimal image quality was first identified based on these assessments. On this optimal monoenergetic image, ROIs were manually delineated separately for the arterial, venous, and delay phases, and subsequently propagated to other spectral CT-derived images using a single-click copy function, including additional monoenergetic images, iodine maps, and effective atomic number maps (Fig. 1C and Supplementary Figure 2). The spectral CT-derived parameters included CT attenuation values at 40, 60, 80, 100, and 120 keV (HU _40 keV_, HU _60 keV_, HU _80keV_, HU _100 keV_, and HU _120 keV_), IC, Zeff, and ED. Dynamic multiphase parameters, including arterial enhancement fraction (AEF), venous enhancement fraction (VEF), delay enhancement fraction (DEF), and interphase difference parameters, were calculated according to the formulas shown in Fig. 2D, where AEF, VEF, and DEF represent arterial, venous, and delay enhancement fractions, respectively.

### Pathology

All pathological slides from the two centers were assessed using the same standardized histopathological criteria. EMVI was diagnosed when tumor cells were unequivocally identified within an endothelium-lined space exhibiting one of the following features: (i) a surrounding rim of smooth muscle or elastin, or (ii) a well-circumscribed tumor nodule immediately adjacent to an artery with intact circumferential elastin [16, 17]. Assessment was performed on hematoxylin and eosin (H&E)-stained and Movat-stained slides, using a multi-head microscope. The assessment was independently performed by two pathologists with 5 and 11 years of experience, who were blinded to the imaging findings and clinical information. In cases of disagreement, the final pathological diagnosis was determined by consensus between the two pathologists.

### Statistical analysis

The data were statistically analyzed using SPSS (version 25.0) and R (version 4.1.0) software. Interobserver agreement between the two pathologists was evaluated using Cohen’s kappa. Interobserver agreement for ROI contours was assessed using the Dice similarity coefficient (DSC), and interobserver consistency of quantitative parameters was evaluated using the intraclass correlation coefficient (ICC). Categorical variables were expressed as frequencies and percentages and compared using the chi-square test. Normally distributed continuous variables are presented as mean±standard deviation and were compared using Student’s t-test, whereas non-normally distributed continuous variables are presented as median and interquartile range (IQR) and were compared using the Mann-Whitney U test. Patients from medical center 1 were randomly divided into training and testing cohorts at a 7:3 ratio. Candidate variables included clinicopathological characteristics and spectral CT-derived parameters. Univariate logistic regression analysis was first performed in the training cohort, and variables with statistical significance were subsequently entered into stepwise multivariable logistic regression analysis to identify independent predictors and construct the nomogram (Fig. 1E). During model construction, multicollinearity was assessed using variance inflation factors (VIFs), and variables with VIF values < 10 were retained. During stepwise model selection, the Akaike information criterion (AIC) was used to compare candidate models. Among models with ΔAIC < 5, the model with the fewest predictors was selected as the final model. Variables included in the final model had no missing values; therefore, no imputation was performed. Model performance was evaluated using receiver operating characteristic (ROC) analysis, calibration curves, the Hosmer-Lemeshow test, and decision curve analysis (DCA) (Fig. 1F). DeLong’s test was used to compare AUCs. *P* < 0.05 was considered statistically significant.

## Results

### Image quality parameters in different monoenergetic CT images

The differences in CT value_−tumor_, SNR, CNR, SD _fat_ and SD _air_ across different monoenergetic images in the arterial, venous, and delay phases were statistically significant (Table 1). In the arterial phase, venous phase, and delay phase, the differences in CT value-tumor, SNR, and CNR were statistically significant, with the highest values observed in the 60 keV images (Supplementary Table 1). In the comparison between the two groups at 80 keV, 100 keV, and 120 keV, there were no statistically significant differences in SD _fat_ and SD _air_, regardless of the arterial phase or venous phase. The average values of computed tomography dose index (CTDIvol), dose length product (DLP), and effective dose (ED) were 14.1 ± 1.3 mGy, 763 ± 115 mGy·cm, and 14.5 ± 1.5 mSv, respectively. The CTDIvol and DLP were provided by the scanner. The ED was calculated using the conversion factor specific to the pelvic region, set at 0.019 mSv/(mGy·cm).

**Table 1:** Image quality parameters of different monoenergetic CT images in the medical center 1 (*n* = 192

	40 keV	60 keV	80 keV	100 keV	120 keV	*P*-value
**arterial phase**						
CT value_−tumor_ (HU)	146.96±42.86	86.82±19.01	64.92±11.60	55.81*±*11.92	54.13*±*22.64	< 0.001***
SNR	8.11*±*3.84	6.12*±*2.18	5.01*±*1.63	4.44*±*1.42	4.44*±*1.42	< 0.001***
CNR	6.52*±*5.94	3.22*±*2.09	1.51*±*1.32	0.74*±*1.65	0.56*±*2.42	< 0.001***
SD _fat_	0.56*±*2.42	13.02*±*3.81	11.96*±*3.34	11.60*±*3.17	11.36*±*3.15	< 0.001***
SD _air_	19.51*±*7.28	13.02*±*3.81	11.35*±*6.08	10.95*±*6.02	10.67*±*5.61	< 0.001***
**venous phase**						
CT value_−tumor_ (HU)	204.12*±*52.82	110.71*±*23.58	77.01*±*13.14	63.00*±*11.88	55.95*±*9.56	< 0.001***
SNR	10.07*±*4.20	7.21*±*2.62	5.52*±*1.64	4.72*±*1.46	4.27*±*1.27	< 0.001***
CNR	9.64*±*5.38	4.53*±*2.55	2.27*±*1.65	1.16*±*1.49	0.58*±*1.23	< 0.001***
SD _fat_	16.40*±*6.23	13.23*±*4.19	12.19*±*3.73	11.84*±*3.54	11.62*±*3.53	< 0.001***
SD _air_	19.59*±*6.43	12.57*±*5.05	10.73*±*4.99	10.25*±*4.92	10.13*±*4.87	< 0.001***
**delay phase**						
CT value_−tumor_ (HU)	173.59*±*35.24	97.44*±*15.54	70.59*±*12.70	59.93*±*15.82	52.38*±*6.53	< 0.001***
SNR	10.04*±*3.82	6.97*±*2.27	5.45*±*1.88	4.70*±*4.20	4.20*±*1.24	< 0.001***
CNR	6.95*±*3.95	3.136*±*2.12	1.50*±*1.60	0.78*±*1.54	0.25*±*1.05	< 0.001***
SD _fat_	15.80*±*5.05	12.96*±*3.65	12.11*±*3.32	11.72*±*3.21	11.54*±*3.18	< 0.001***
SD _air_	20.19*±*7.36	13.12*±*6.19	11.17*±*6.06	10.63*±*5.92	10.49*±*5.84	< 0.001***

SNR: Signal-to-Noise Ratio; CNR: Contrast-to-Noise Ratio; SD: Standard Deviation

*: *P*-value < 0.05, **: *P*-value < 0.01, ***: *P*-value < 0.001

Therefore, subjective scoring of overall image quality and tumor visibility was subsequently performed on 60 keV and 80 keV images. Interobserver consistency analysis showed an ICC of 0.92 between the two observers. In the arterial and venous phase, tumor visibility scores differed significantly between 60 keV and 80 keV images, whereas overall image quality scores showed no significant differences. In the delay phase, both the CT image and tumor scoring showed statistically significant differences between the 60 keV and 80 keV groups, with higher subjective scores for the 60 keV images (Table 2).

**Table 2:** Comparison of subjective scores between 60 keV and 80 keV images

	CT image		*P*-value	tumor			*P*-value
	60 keV	80 keV		60 keV	80 keV		
arterial phase	4.76*±*0.46	4.75*±*0.47	0.827	4.41*±*0.65		4.19*±*0.67	< 0.001***
venous phase	4.85*±*0.37	4.77*±*0.42	0.059	4.62*±*0.58		4.42*±*0.67	0.002**
delay phase	4.82*±*0.40	4.72*±*0.48	0.030*	4.51*±*0.62		4.17*±*0.63	< 0.001***

*: *P*-value < 0.05, **: *P*-value < 0.01, ***: *P*-value < 0.001

### Spectral CT-derived parameters and dynamic multiphase spectral parameters

Interobserver agreement for histopathological EMVI assessment was excellent, with a Cohen’s kappa value of 0.83. Based on the previous results regarding image quality, this study delineated the ROI on the 60 keV images to measure the spectral CT-derived parameters. The ROI contour agreement between the two radiologists was good, with a DSC of 0.81, indicating acceptable interobserver reproducibility of manual ROI delineation. Interobserver consistency analysis showed that all ICC values between the two observers were above 0.90 (Supplementary Table 2).

In the triple-phase enhancement, there were no statistically significant differences in the original spectral CT-derived parameters (including monoenergetic CT values, k, IC, NIC, Zeff, and ED) between the EMVI positive and EMVI negative groups (Supplementary Table 3). No significant differences in clinical features or TNM stage were observed between the EMVI positive and EMVI negative groups in medical center 1. Conventional CT-based EMVI assessment showed an accuracy of 67.2% for predicting pathological EMVI, as shown in Supplementary Table 3.

Dynamic multiphase spectral parameters were compared between EMVI positive and EMVI negative groups. There were statistically significant differences in ΔHU40 _(delay−arterial)_, ΔHU60 _(delay−arterial)_, ΔIC _(delay−arterial)_, DEF, and ΔZeff _(venous−arterial)_ between the EMVI positive and EMVI negative groups, with higher values in the positive group (Table 3 and Supplementary Figure 3).

**Table 3:** Comparison of spectral CT parameters between EMVI positive and EMVI negative groups in the medical center 1 (*n* = 192)

Spectral CT parameters	EMVI		*P*-value
	Positive group (*n* = 71)	Negative group (*n* = 121)	
ΔHU 40 _(venous−arterial)_^#^	64.78*±*64.82	52.69*±*56.07	0.175
ΔHU40 _(venous−delay)_^#^	29.04*±*49.12	31.40*±*36.06	0.703
ΔHU40 _(delay−arterial)_^#^	60.49*±*29.75	30.30*±*18.66	< 0.001***
ΔHU60 _(venous−arterial)_^#^	26.65*±*29.42	22.29*±*23.08	0.256
ΔHU60 _(venous−delay)_^#^	12.03*±*20.28	14.01*±*16.35	0.462
ΔHU60 _(delay−arterial)_^#^	29.53*±*12.73	14.90*±*9.86	< 0.001***
ΔHU80 _(venous−arterial)_^#^	13.58*±*16.39	11.21*±*13.01	0.272
ΔHU80 _(venous−delay)_^#^	60.68*±*11.84	6.26*±*14.74	0.841
ΔHU80 _(delay−arterial)_^#^	6.90*±*15.02	4.95*±*15.83	0.402
ΔHU100 _(venous−arterial)_^#^	8.04*±*12.68	6.70*±*15.41	0.536
ΔHU100 _(venous−delay)_^#^	2.72*±*14.65	3.27*±*19.49	0.837
ΔHU100 _(delay−arterial)_^#^	5.32*±*17.81	3.43*±*20.57	0.519
ΔIC _(venous−arterial)_^##^	0.75*±*0.70	0.57*±*0.60	0.070
ΔIC _(venous−delay)_^##^	-1.22*±*0.41	-1.15*±*0.36	0.208
ΔIC _(delay−arterial)_^##^	0.58*±*0.32	0.33*±*0.20	< 0.001***
AEF	0.70*±*0.35	0.74*±*0.31	0.399
VEF	1.21*±*0.37	1.24*±*0.35	0.489
DEF	0.81*±*0.25	0.65*±*0.21	< 0.001***
ΔZeff _(venous−arterial)_	0.49*±*0.30	0.23*±*0.24	< 0.001***
ΔZeff _(venous−delay)_	0.14*±*0.24	0.13*±*0.23	0.790
ΔZeff _(delay−arterial)_	-0.24*±*0.27	-0.15*±*0.32	0.061
ΔED _(venous−arterial)_	-0.16*±*0.56	-0.14*±*0.63	0.846
ΔED _(venous−delay)_	0.11*±*0.54	0.13*±*0.62	0.841
ΔED _(delay−arterial)_	-0.05*±*0.63	-0.01*±*0.62	0.690

^#^: parameters measured in Hounsfield units (HU); ^##^: parameters measured in mg/mL

*: *P*-value < 0.05, **: *P*-value < 0.01, ***: *P*-value < 0.001

### Predictive model construction and validation

The cases from medical center 1 were randomly divided into a training cohort and a testing cohort at a ratio of 7:3. In the training cohort, 134 patients were included, comprising 50 EMVI-positive and 84 EMVI-negative patients. The testing cohort consisted of 58 patients, including 21 EMVI-positive and 37 EMVI-negative patients.

In the training set, dynamic multiphase spectral CT‑derived parameters with *P* < 0.05 in univariate and multivariable logistic regression analysis were used (Table 4). Subsequently, these candidate predictors were entered into backward stepwise multivariable logistic regression. The combined model was presented as a nomogram (Fig. 2A). The predicted probability of EMVI positivity was calculated as follows: logit(P) = -6.642་0.063 × ΔHU60 keV _(delay−arterial)_་0.038 × ΔHU40 keV _(delay−arterial)_ ་0.671 × ΔIC _(delay−arterial)_ + 2.830×DEF་1.827 × ΔZeff _(venous−arterial)_.

**Table 4:** Univariable and multivariable logistic regression analysis of the spectral CT parameters derived from colorectal cancer for predicting EMVI in the training cohort (*n* = 134)

DECT parameters	Univariate logistic regression			Multivariate logistic regression		
	OR	95%CI	*P*-value	OR	95%CI	*P*-value
ΔHU40 _(delay−arterial)_	5.36	(3.06, 10.51)	< 0.001***	2.74	(1.12, 7.84)	0.029*
ΔHU60 _(delay−arterial)_	4.06	(2.50, 7.12)	< 0.001***	3.44	(1.56, 8.83)	< 0.002**
ΔIC _(delay−arterial)_	3.20	(2.02, 5.49)	< 0.001***	2.91	(1.11, 8.42)	0.030*
DEF	2.28	(1.51, 3.66)	< 0.001*	3.40	(1.85, 5.49)	< 0.001***
ΔZeff _(venous−arterial)_	4.37	(2.46, 8.67)	< 0.001*	5.27	(2.11, 17.52)	< 0.001***

*: *P*-value < 0.05, **: *P*-value < 0.01, ***: *P*-value < 0.001

**Fig. 3:**
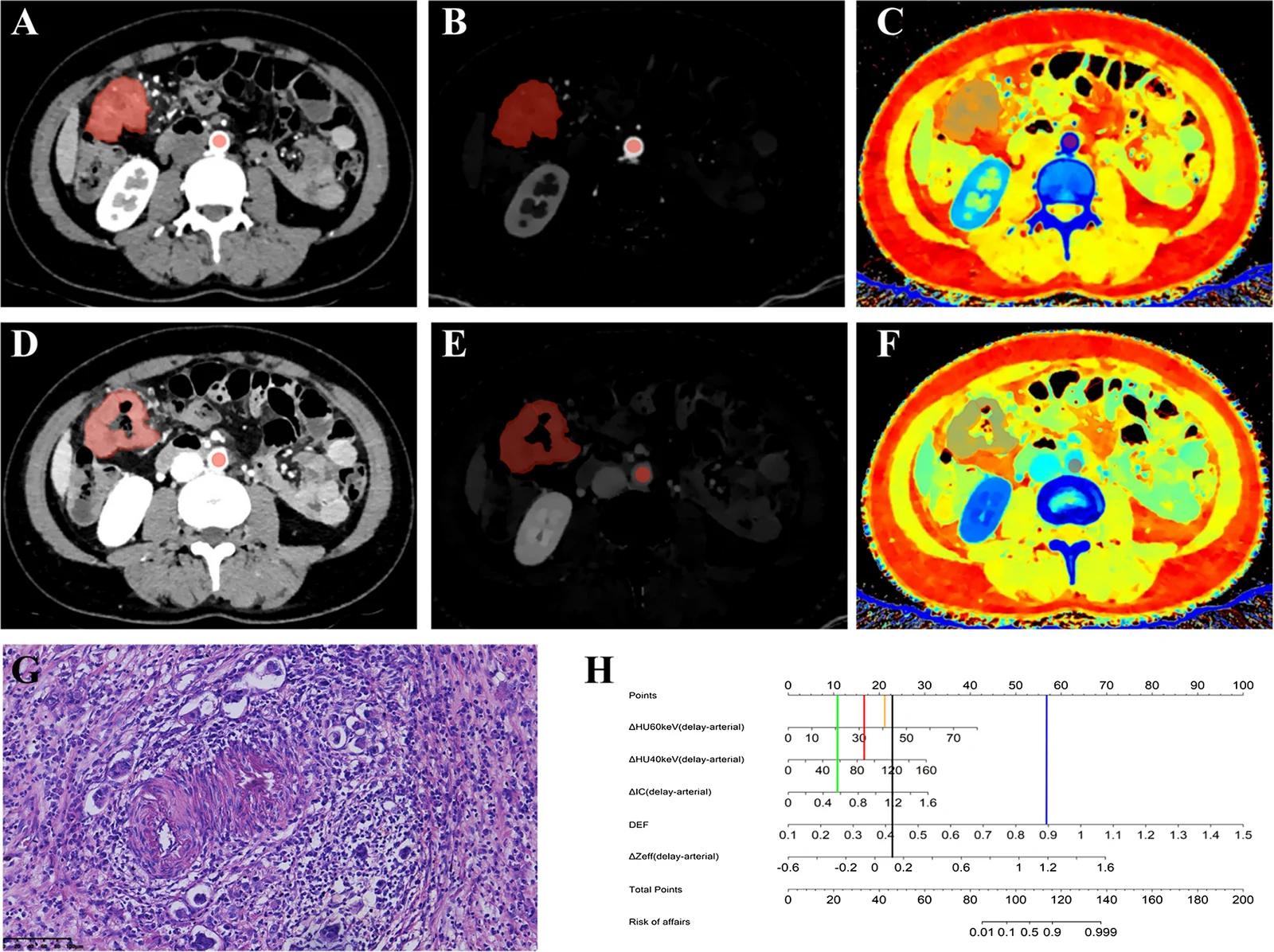
A 58-year-old female patient with colon cancer. (**A**), (**B**), and (**C**) show the arterial-phase 60 keV VMI, iodine map, and Zeff map, respectively. (**D**), (**E**), and (**F**) show the delay-phase 60 keV VMI, iodine map, and Zeff map, respectively. (**G**) shows the H&E-stained pathological image, indicating EMVI positivity. (**H**) shows the nomogram score; the total score of 129 points corresponds to a predicted probability of EMVI positive greater than 90%

The ROC curve analysis of the five selected predictors and the model is shown in Fig. 2B for the training, testing, and external validation sets. The AUCs of the model in the training, testing, and external validation cohorts were 0.93, 0.91, and 0.84, respectively (Table 5; Fig. 2B). The optimal cut-off value of the nomogram was determined as 0.408 using the Youden index in the training cohort. Patients with a predicted probability ≥ 0.408 were classified as EMVI-positive. The Hosmer-Lemeshow test yielded P values of 0.45, 0.39, and 0.18 in the training, testing, and external validation cohorts, respectively, indicating satisfactory calibration across the datasets (Fig. 2C). The calibration intercepts were 0.304, 0.257, and 0.213 in the training, internal testing, and external validation cohorts, respectively, and the corresponding calibration slopes were 1.287, 1.250, and 0.992, respectively. DCA showed that the EMVI-predicting nomogram yielded a greater net benefit than the individual risk factors across the training, testing, and external validation cohorts, supporting its clinical applicability and generalizability (Fig. 2D). A representative patient case is shown in Fig. 3.

**Table 5:** Risk factors and combined model of spectral CT for predicting EMVI from colon cancer

	AUC (95%CI)	Sen	Spe		Acc	PPV	NPV	Delong test
**Training cohort** **(** ***n*** ** = 134)**								
Model	0.93 (0.89, 0.97)	0.86	0.87		0.87	0.80	0.91	NA
ΔHU40 _(delay−arterial)_	0.84 (0.78, 0.91)	0.82	0.73		0.71	0.64	0.87	< 0.001***
ΔHU60 _(delay−arterial)_	0.81 (0.73, 0.88)	0.76	0.74		0.75	0.63	0.84	< 0.001***
ΔIC _(delay−arterial)_	0.77 (0.67, 0.85)	0.82	0.61		0.67	0.55	0.86	< 0.001***
DEF	0.67 (0.58, 0.77)	0.68	0.67		0.67	0.55	0.78	< 0.001***
ΔZeff _(venous−arterial)_	0.77 (0.69, 0.85)	0.68	0.73		0.71	0.60	0.79	< 0.001***
**Testing cohort (n = 58)**								
Model	0.91 (0.83, 0.98)	0.85	0.89		0.81	0.83	0.92	NA
ΔHU40 _(delay−arterial)_	0.76 (0.63, 0.89)	0.62	0.68		0.66	0.53	0.75	0.006**
ΔHU60 _(delay−arterial)_	0.85 (0.75, 0.95)	0.57	0.86		0.76	0.71	0.77	0.024*
ΔIC _(delay−arterial)_	0.71 (0.56, 0.85)	0.71	0.46		0.55	0.44	0.74	0.003**
DEF	0.66 (0.52, 0.80)	0.48	0.70		0.62	0.49	0.69	0.005**
ΔZeff _(venous−arterial)_	0.68 (0.54, 0.82)	0.48	0.76		0.66	0.55	0.71	< 0.001***
**Validation cohort** **(n = 43)**								
Model	0.84 (0.74, 0.93)	0.83	0.81		0.88	0.72	0.90	NA
ΔHU40 _(delay−arterial)_	0.79 (0.59–0.97)	0.69	0.87		0.81	0.76	0.83	0.032*
ΔHU60 _(delay−arterial)_	0.77 (0.59, 0.96)	0.62	0.80		0.74	0.65	0.78	0.017*
ΔIC _(delay−arterial)_	0.74 (0.57, 0.91)	0.69	0.50		0.56	0.46	0.74	0.028*
DEF	0.70 (0.51, 0.88)	0.46	0.73		0.65	0.50	0.69	0.005**
ΔZeff _(venous−arterial)_	0.81 (0.67, 0.92)	0.69	0.77		0.75	0.65	0.81	0.079

Sen: sensitivity; Spe: specificity; Acc: accuracy; PPV: positive predictive value; NPV: negative predictive

*: *P*-value < 0.05, **: *P*-value < 0.01, ***: *P*-value < 0.001. NA: not applicable

## Discussion

This study demonstrates that spectral CT not only enhances image quality in colon cancer but also enables non-invasive preoperative prediction of EMVI status. Among the various contrast-enhanced phases, the 60 keV VMIs provide superior delineation of tumor boundaries compared with conventional CT, thereby facilitating a more accurate characterization of intratumoral heterogeneity. A nomogram model based on dynamic multiphase spectral CT-derived parameters, including ΔHU40 _(delay−arterial)_, ΔHU60 _(delay−arterial)_, ΔIC _(delay−arterial)_, DEF, and ΔZeff _(venous−arterial)_, showed excellent performance in non-invasive prediction of EMVI status in the surgical region, with an AUC of 0.84 in an independent external validation cohort. Compared with conventional CT-EMVI assessment, which showed limited sensitivity and overall discriminative ability, the spectral CT-based nomogram demonstrated higher predictive performance.

In this study, 60 keV VMI showed better image quality for colon cancer lesion visualization among the evaluated triphasic contrast-enhanced spectral CT images. Objective metrics, including tumor CT value, CNR, and SNR, together with subjective image quality scores, suggested that 60 keV may provide improved lesion conspicuity compared with other energy levels. Racine et al. reported that liver lesions in abdominal phantoms showed high detectability at 65 and 70 keV VMIs [18], which is broadly consistent with our findings. In contrast, another study on colorectal cancer reported that 40 keV VMI achieved the highest quantitative image parameters and improved tumor detection [15]. This discrepancy may be related to differences in tumor location and surrounding anatomy, as colon cancers are mainly located in the abdominal cavity, whereas rectal cancers are situated in the pelvis. At low keV levels, iodine contrast is increased, but image noise may also be amplified [11]. Therefore, VMI at 60 keV may provide a reasonable balance between contrast enhancement and image noise reduction for contrast-enhanced CT imaging of colon cancer.

In this study, pathological T stage was not significantly associated with EMVI status (*P* = 0.067), although the EMVI positive group showed a higher proportion of T4 tumors (57.7% vs. 43.0%). This trend is biologically plausible and consistent with previous reports [19], suggesting that deeper tumor invasion may increase the risk of EMVI. However, as T stage did not reach statistical significance, it was not included in the final model. Dynamic multiphase spectral CT parameters may provide additional information related to tumor angiogenesis, microvascular perfusion, and hemodynamic characteristics. Although their value for EMVI prediction has been reported in rectal cancer [20], evidence in colon cancer remains limited.

Among spectral CT-derived parameters, this study found no significant differences in tumor VMI related CT values, IC, Zeff, or ED between EMVI-positive and EMVI-negative groups, indicating the limitations of single-phase enhancement. We then analyzed dynamic multiphase spectral CT-derived parameters and observed that ΔHU40 _(delay−arterial)_, ΔHU60 _(delay−arterial)_, ΔIC _(delay−arterial)_, DEF, and ΔZeff _(venous−arterial)_ were all higher in the EMVI-positive group. ΔHU40 _(delay−arterial)_ and ΔHU60 _(delay−arterial)_ represent changes in CT attenuation between the delay and arterial phases at 40 keV and 60 keV VMI, respectively, and may reflect dynamic tumor enhancement patterns. Colon cancer, similar to other malignancies, may show rapid contrast wash-in and wash-out related to increased and disorganized intratumoral vasculature. Previous studies have reported associations between tumor invasiveness, microvascular density (MVD), and tumor blood volume [21, 22]. Therefore, larger delay-arterial differences in CT attenuation may be associated with richer tumor vascularity and faster contrast clearance. The lack of meaningful differences between arterial, venous and delay phases in this study may be because contrast clearance is less pronounced during the venous phase than during the delay phase.

Previous studies have demonstrated that IC is more sensitive than conventional contrast-enhanced CT in reflecting lesion enhancement [23, 24]. ΔIC _(delay−arterial)_ may provide a more direct assessment of iodine distribution within the tumor and reduce the influence of misregistration between non-contrast and enhanced images [25]. DEF may reflect dynamic changes in tumor perfusion and may be indirectly associated with MVD and extracellular matrix alterations. Because angiogenesis and extracellular matrix remodeling are involved in tumor progression, invasion, and metastasis [26], the higher DEF in the EMVI positive group may indicate more aggressive tumor biological behavior, which is consistent with previous study [27].

Zeff reflects the average atomic number of compounds composed of different tissue constituents [28] and can help differentiate materials with similar CT attenuation but different compositions [23, 25]. Zeff is positively correlated with tissue density [29], with higher values corresponding to denser tissue. Previous studies have associated elevated Zeff with lymph node metastasis prediction [30], and peritoneal metastases from colorectal cancer with poor treatment response have been reported to show densely packed tumor cells and higher Zeff values [31], which is consistent with our findings.

Incorporating the aforementioned features, we further constructed a predictive model that enhanced the preoperative CT-based assessment of EMVI in colon cancer. To improve the generalizability of the model, an independent external validation cohort from medical center 2 was included. The predictive model demonstrated robust performance across the training set, internal testing set, and external validation set. Compared with previously reported conventional CT models for predicting EMVI, which demonstrated a sensitivity of 44% and a specificity of 77% [10], the nomogram developed in this study based on dynamic multiphase spectral CT parameters achieved both sensitivity and specificity exceeding 80%. Although the AUC decreased in the external validation cohort, this may be mainly attributed to differences in patient composition between centers, as a consistent scanning protocol was used across the participating centers. Nevertheless, the AUC of 0.84 in the independent external validation cohort still indicates good predictive performance and supports the potential generalizability of the model.

The tumor-based ROI strategy used in this study was designed to capture intratumoral dynamic enhancement patterns and quantitative spectral CT features, which may provide indirect imaging information related to EMVI status. In colon cancer, standardized delineation of peritumoral or perivascular regions on CT images remains challenging because of the complex pericolonic anatomy and potential interference from mesenteric fat, inflammatory changes, lymph nodes, and partial volume effects. Therefore, peritumoral and perivascular regions were not included in the present analysis. Future studies incorporating these regions may provide a more comprehensive characterization of the imaging features related to the peritumoral and perivascular microenvironment of EMVI.

However, our study has several limitations: (1) No sampling, matching, or artificial balancing procedure was performed, and the original distribution of EMVI status in the real-world cohort was retained. Although the imbalance between EMVI-positive and EMVI-negative cases was not extreme, potential effects of class imbalance on model training and performance estimation cannot be completely excluded. (2) Because stepwise logistic regression was used for variable selection, the possibility of optimistic performance estimation cannot be completely excluded. Future studies should further adopt bootstrap resampling, cross-validation, or penalized regression methods to improve model robustness and reduce the risk of overfitting. (3) Only intratumoral ROIs were measured, as peritumoral structures such as lymph nodes and partial volume effects may affect measurement reliability, however, peritumoral features may reflect the tumor microenvironment and should be further investigated in future studies. (4) The sample size of this study was relatively small, particularly in the external validation cohort; therefore, further validation in larger multicenter cohorts is needed.

## Conclusion

In conclusion, 60 keV VMI effectively enhances image quality, providing a robust basis for precise tumor delineation. Moreover, the nomogram integrating dynamic multiphase parameters (ΔHU40 _(delay−arterial)_, ΔHU60 _(delay−arterial)_, ΔIC _(delay−arterial)_, DEF, and ΔZeff _(venous−arterial)_) exhibits high clinical value for preoperative assessment of EMVI in colon cancer, offering reliable imaging-based guidance for individualized diagnosis and therapeutic planning.

## Supplementary Information

Below is the link to the electronic supplementary material.

Supplementary Material 1

## Data Availability

The data and materials of this study are available from the corresponding author on reasonable request.

## Abbreviations

EMVI: Extramural venous invasion
VMI: Virtual monoenergetic imaging
SNR: Signal-to-noise ratio
CNR: Contrast-to-noise ratio
IC: Iodine concentration
Zeff: Effective atomic number
AUC: Area under the curve
SD: Standard deviation
ROI: Region of interest
ROC: Receiver operating characteristic
ICC: Intraclass correlation coefficient
MVD: Microvessel density
