# Supplementary material for: Development of a nomogram for predicting extramural venous invasion in colon cancer using spectral CT multiparameter imaging
Source: Cancer Imaging. 2026 Jul 31;26:126. doi: 10.1186/s40644-026-01098-6 (PMC13576261; doi:10.1186/s40644-026-01098-6)
Supplement: Supplementary file 1 [file 40644_2026_1098_MOESM1_ESM.docx]

**Supplementary materials**

**Supplementary Table 1. Image quality parameters of different electron energy levels in monoenergetic image (T-test or Mann-Whitney U test).**

|  | 40keV | 60keV | 80keV | 100keV | 120keV | P-value | | | | | | | | | |
| --- | --- | --- | --- | --- | --- | --- | --- | --- | --- | --- | --- | --- | --- | --- | --- |
|  |  |  |  |  |  | 40keV vs. 60keV | 40keV vs. 80keV | 40keV vs. 100keV | 40keV vs. 120keV | 60keV vs. 80keV | 60keV vs. 100keV | 60keV vs. 120keV | 80keV vs. 100keV | 80keV vs. 120keV | 100keVvs. 120keV |
| **Arterial phase** |  |  |  |  |  |  |  |  |  |  |  |  |  |  |  |
| CT value-_tumor_ (HU) | 146.96$\pm$42.86 | 86.82$\pm$19.01 | 64.92$\pm$11.60 | 55.81$\pm$11.92 | 54.13$\pm$22.64 | <0.001 | <0.001 | <0.001 | <0.001 | <0.001 | <0.001 | <0.001 | <0.001 | <0.001 | 0.502 |
| SNR | 8.11$\pm$3.84 | 6.12$\pm$2.18 | 5.01$\pm$1.63 | 4.44$\pm$1.42 | 4.44$\pm$1.42 | <0.001 | <0.001 | <0.001 | <0.001 | <0.001 | <0.001 | <0.001 | 0.020 | 0.009 | 0.780* |
| CNR | 6.52$\pm$5.94 | 3.22$\pm$2.09 | 1.51$\pm$1.32 | 0.74$\pm$1.65 | 0.56$\pm$2.42 | <0.001 | <0.001 | <0.001 | <0.001 | <0.001 | <0.001 | <0.001 | 0.018 | 0.004 | 0.587* |
| SD_fat_ | 0.56$\pm$2.42 | 13.02$\pm$3.81 | 11.96$\pm$3.34 | 11.60$\pm$3.17 | 11.36$\pm$3.15 | <0.001 | <0.001 | <0.001 | <0.001 | 0.042 | 0.013 | 0.003 | 0.527* | 0.284* | 0.661* |
| SD_air_ | 19.51$\pm$7.28 | 13.02$\pm$3.81 | 11.35$\pm$6.08 | 10.95$\pm$6.02 | 10.67$\pm$5.61 | <0.001 | <0.001 | <0.001 | <0.001 | 0.005 | 0.001 | <0.001 | 0.528* | 0.287* | 0.665* |
| **Venous phase** |  |  |  |  |  |  |  |  |  |  |  |  |  |  |  |
| CT value-_tumor_ (HU) | 204.12$\pm$52.82 | 110.71$\pm$23.58 | 77.01$\pm$13.14 | 63.00$\pm$11.88 | 55.95$\pm$9.56 | <0.001 | <0.001 | <0.001 | <0.001 | <0.001 | <0.001 | <0.001 | <0.001 | <0.001 | 0.012 |
| SNR | 10.07$\pm$4.20 | 7.21$\pm$2.62 | 5.52$\pm$1.64 | 4.72$\pm$1.46 | 4.27$\pm$1.27 | <0.001 | <0.001 | <0.001 | <0.001 | <0.001 | <0.001 | <0.001 | 0.002 | <0.001 | 0.076* |
| CNR | 9.64$\pm$5.38 | 4.53$\pm$2.55 | 2.27$\pm$1.65 | 1.16$\pm$1.49 | 0.58$\pm$1.23 | <0.001 | <0.001 | <0.001 | <0.001 | <0.001 | <0.001 | <0.001 | <0.001 | <0.001 | 0.048 |
| SD_fat_ | 16.40$\pm$6.23 | 13.23$\pm$4.19 | 12.19$\pm$3.73 | 11.84$\pm$3.54 | 11.62$\pm$3.53 | <0.001 | <0.001 | <0.001 | <0.001 | 0.021 | 0.002 | <0.001 | 0.436* | 0.199* | 0.613* |
| SD_air_ | 19.59$\pm$6.43 | 12.57$\pm$5.05 | 10.73$\pm$4.99 | 10.25$\pm$4.92 | 10.13$\pm$4.87 | <0.001 | <0.001 | <0.001 | <0.001 | 0.001 | <0.001 | <0.001 | 0.379* | 0.270* | 0.824* |
| **Delay phase** |  |  |  |  |  |  |  |  |  |  |  |  |  |  |  |
| CT value-_tumor_ (HU) | 173.59$\pm$35.24 | 97.44$\pm$15.54 | 70.59$\pm$12.70 | 59.93$\pm$15.82 | 52.38$\pm$6.53 | <0.001 | <0.001 | <0.001 | <0.001 | <0.001 | <0.001 | <0.001 | <0.001 | <0.001 | <0.001 |
| SNR | 10.04$\pm$3.82 | 6.97$\pm$2.27 | 5.45$\pm$1.88 | 4.70$\pm$4.20 | 4.20$\pm$1.24 | <0.001 | <0.001 | <0.001 | <0.001 | <0.001 | <0.001 | <0.001 | <0.001 | 0.002 | 0.037 |
| CNR | 6.95$\pm$3.95 | 3.136$\pm$2.12 | 1.50$\pm$1.60 | 0.78$\pm$1.54 | 0.25$\pm$1.05 | <0.001 | <0.001 | <0.001 | <0.001 | <0.001 | <0.001 | <0.001 | 0.002 | <0.001 | 0.022 |
| SD_fat_ | 15.80$\pm$5.05 | 12.96$\pm$3.65 | 12.11$\pm$3.32 | 11.72$\pm$3.21 | 11.54$\pm$3.18 | <0.001 | <0.001 | <0.001 | <0.001 | 0.026 | 0.001 | <0.001 | 0.313* | 0.140* | 0.641* |
| SD_air_ | 20.19$\pm$7.36 | 13.12$\pm$6.19 | 11.17$\pm$6.06 | 10.63$\pm$5.92 | 10.49$\pm$5.84 | <0.001 | <0.001 | <0.001 | <0.001 | 0.002 | <0.001 | <0.001 | 0.402* | 0.293* | 0.831* |

SNR: signal-to-noise ratio; CNR: contrast-to-noise ratio; SD: standard deviation.

*: P-value>0.05.

**Supplementary Table 2. Interobserver agreement for ROI-derived spectral CT parameters**

|  | IC | ED | Zeff | CT value-_40keV_ | CT value-_60keV_ | CT value-_80keV_ | CT value-_100keV_ | CT value-_120keV_ |
| --- | --- | --- | --- | --- | --- | --- | --- | --- |
| ICC-AP | 0.91 | 0.90 | 0.95 | 0.92 | 0.94 | 0.91 | 0.93 | 0.92 |
| ICC-VP | 0.95 | 0.94 | 0.91 | 0.93 | 0.96 | 0.94 | 0.92 | 0.91 |
| ICC-DP | 0.93 | 0.94 | 0.95 | 0.92 | 0.93 | 0.91 | 0.93 | 0.96 |

IC: iodine concentration; ED: electron density; Zeff: effective atomic number; ICC: intraclass correlation coefficient; AP: arterial phase;

VP: venous phase; DP: delay phase.

**Supplementary Table 3. Comparison of clinical features and spectral CT parameters between EMVI-positive and EMVI-negative groups in the medical center 1.**

|  | EMVI | | P-value |
| --- | --- | --- | --- |
|  | positive group (n=71) | negative group (n=121) |  |
| gender |  |  | 0.891 |
| female | 31(43.7%) | 54 (44.6%) |  |
| male | 40 (56.3%) | 67 (55.4%) |  |
| age | 62.77$\text{±}$13.06 | 62.01$\text{±}$13.80 | 0.706 |
| Tumor location |  |  | 0.885 |
| right | 28 (39.4%) | 49 (40.5%) |  |
| left | 43 (60.6%) | 72 (59.5%) |  |
| CT-EMVI |  |  | <0.001*** |
| positive | 36 (50.7%) | 28 (23.1%) |  |
| negative | 35 (49.3%) | 93 (76.9%) |  |
| Pathological T stage |  |  | 0.067 |
| Tis | 0 | 0 |  |
| 1 | 0 | 1 (0.8%) |  |
| 2 | 0 | 10 (8.3%) |  |
| 3 | 30 (42.3%) | 58 (47.9%) |  |
| 4 | 41 (57.7%) | 52 (43.0%) |  |
| Pathological N stage |  |  | 0.272 |
| 0 | 33 (46.5%) | 73 (60.3%) |  |
| 1 | 28 (39.4%) | 37 (30.6%) |  |
| 2 | 10 (14.1%) | 11 (9.1%) |  |
| **Arterial phase** |  |  |  |
| IC | 1.31$\text{±}$0.48 | 1.18$\text{±}$0.47 | 0.050 |
| ED | 104.29$\text{±}$0.59 | 104.22$\text{±}$0.63 | 0.442 |
| Zeff | 8.03$\text{±}$0.25 | 7.97$\text{±}$0.25 | 0.060 |
| CT value-_40keV_ (HU) | 150.59$\text{±}$44.87 | 140.76$\text{±}$38.72 | 0.126 |
| CT value-_60keV_ (HU) | 88.24$\text{±}$18.53 | 84.38$\text{±}$19.70 | 0.175 |
| CT value-_80keV_ (HU) | 65.69$\text{±}$10.83 | 63.60$\text{±}$12.79 | 0.229 |
| CT value-_100keV_ (HU) | 56.606$\text{±}$12.49 | 54.45$\text{±}$10.84 | 0.227 |
| CT value-_120keV_ (HU) | 55.50$\text{±}$26.10 | 51.80$\text{±}$14.91 | 0.275 |
| **Venous phase** |  |  |  |
| IC | 1.89$\text{±}$0.56 | 1.92$\text{±}$0.70 | 0.695 |
| ED | 104.43$\text{±}$0.61 | 104.38$\text{±}$0.58 | 0.565 |
| Zeff | 8.31$\text{±}$0.26 | 8.34$\text{±}$0.34 | 0.585 |
| CT value-_40keV_ (HU) | 203.28$\text{±}$48.25 | 205.55$\text{±}$60.31 | 0.787 |
| CT value-_60keV_ (HU) | 110.53$\text{±}$21.11 | 111.03$\text{±}$27.44 | 0.895 |
| CT value-_80keV_ (HU) | 76.90$\text{±}$11.87 | 77.18$\text{±}$15.16 | 0.889 |
| CT value-_100keV_ (HU) | 63.30$\text{±}$12.61 | 62.49$\text{±}$10.58 | 0.647 |
| CT value-_120keV_ (HU) | 5.68$\text{±}$7.42 | 56.41$\text{±}$12.44 | 0.612 |
| **Delay phase** |  |  |  |
| IC | 1.55$\text{±}$0.35 | 1.61$\text{±}$0.44 | 0.330 |
| ED | 104.30$\text{±}$0.55 | 104.27$\text{±}$0.59 | 0.703 |
| Zeff | 8.19$\text{±}$0.24 | 8.20$\text{±}$0.24 | 0.650 |
| CT value-_40keV_ (HU) | 171.88$\text{±}$31.94 | 176.51$\text{±}$40.33 | 0.410 |
| CT value-_60keV_ (HU) | 96.52$\text{±}$14.10 | 98.99$\text{±}$17.73 | 0.318 |
| CT value-_80keV_ (HU) | 70.64$\text{±}$13.74 | 70.50$\text{±}$10.74 | 0.943 |
| CT value-_100keV_ (HU) | 60.03$\text{±}$16.62 | 59.77$\text{±}$14.46 | 0.911 |
| CT value-_120keV_ (HU) | 52.28$\text{±}$6.08 | 52.55$\text{±}$7.28 | 0.782 |

EMVI: extramural venous invasion; IC: iodine concentration; ED: electron density; Zeff: effective atomic number.

*: P-value<0.05, **: P-value<0.01, ***: P-value<0.001.

**Supplementary Figure 1.** The ROI of image quality parameters in monoenergetic CT images.

**Supplementary Figure 2.** ROIs of the colon tumor and artery were manually delineated on the optimal monoenergetic image (A). The ROIs were then copied to the iodine map (B) and the effective atomic number map (C).

**Supplementary Figure 3.** Violin scatter plots illustrate the distribution of spectral CT–derived parameters between EMVI-positive and EMVI-negative groups. (A) ΔHU40 _(delay-arterial)_, (B) ΔHU60 _(delay-arterial)_, (C) ΔIC _(delay-arterial)_, (D) DEF, and (E) ΔZeff _(venous-arterial)_.
